# Short-Range Forecasting of COVID-19 During Early Onset at County, Health District, and State Geographic Levels Using Seven Methods: Comparative Forecasting Study

**DOI:** 10.2196/24925

**Published:** 2021-03-23

**Authors:** Christopher J Lynch, Ross Gore

**Affiliations:** 1 Virginia Modeling, Analysis, and Simulation Center Old Dominion University Suffolk, VA United States

**Keywords:** coronavirus disease 2019, COVID-19, infectious disease, emerging outbreak, forecasting, modeling and simulation, public health, modeling disease outbreaks

## Abstract

**Background:**

Forecasting methods rely on trends and averages of prior observations to forecast COVID-19 case counts. COVID-19 forecasts have received much media attention, and numerous platforms have been created to inform the public. However, forecasting effectiveness varies by geographic scope and is affected by changing assumptions in behaviors and preventative measures in response to the pandemic. Due to time requirements for developing a COVID-19 vaccine, evidence is needed to inform short-term forecasting method selection at county, health district, and state levels.

**Objective:**

COVID-19 forecasts keep the public informed and contribute to public policy. As such, proper understanding of forecasting purposes and outcomes is needed to advance knowledge of health statistics for policy makers and the public. Using publicly available real-time data provided online, we aimed to evaluate the performance of seven forecasting methods utilized to forecast cumulative COVID-19 case counts. Forecasts were evaluated based on how well they forecast 1, 3, and 7 days forward when utilizing 1-, 3-, 7-, or all prior–day cumulative case counts during early virus onset. This study provides an objective evaluation of the forecasting methods to identify forecasting model assumptions that contribute to lower error in forecasting COVID-19 cumulative case growth. This information benefits professionals, decision makers, and the public relying on the data provided by short-term case count estimates at varied geographic levels.

**Methods:**

We created 1-, 3-, and 7-day forecasts at the county, health district, and state levels using (1) a naïve approach, (2) Holt-Winters (HW) exponential smoothing, (3) a growth rate approach, (4) a moving average (MA) approach, (5) an autoregressive (AR) approach, (6) an autoregressive moving average (ARMA) approach, and (7) an autoregressive integrated moving average (ARIMA) approach. Forecasts relied on Virginia’s 3464 historical county-level cumulative case counts from March 7 to April 22, 2020, as reported by *The New York Times*. Statistically significant results were identified using 95% CIs of median absolute error (MdAE) and median absolute percentage error (MdAPE) metrics of the resulting 216,698 forecasts.

**Results:**

The next-day MA forecast with 3-day look-back length obtained the lowest MdAE (median 0.67, 95% CI 0.49-0.84, *P*<.001) and statistically significantly differed from 39 out of 59 alternatives (66%) to 53 out of 59 alternatives (90%) at each geographic level at a significance level of .01. For short-range forecasting, methods assuming stationary means of prior days’ counts outperformed methods with assumptions of weak stationarity or nonstationarity means. MdAPE results revealed statistically significant differences across geographic levels.

**Conclusions:**

For short-range COVID-19 cumulative case count forecasting at the county, health district, and state levels during early onset, the following were found: (1) the MA method was effective for forecasting 1-, 3-, and 7-day cumulative case counts; (2) exponential growth was not the best representation of case growth during early virus onset when the public was aware of the virus; and (3) geographic resolution was a factor in the selection of forecasting methods.

## Introduction

The scientific community responded quickly to the global outbreak following COVID-19’s identification in December of 2019 [1,2]. Numerous platforms and studies have been created to forecast the spread of the pandemic and meet the need for intervention measures in support of public health and awareness [1,3-5]. Many forecasting efforts focused on the long-term identification of COVID-19 and the flattening of and getting over the curve [3,4,6]. Forecasts assist in identifying and evaluating long-term preventions. Short-range forecasts provide benefit by supporting local understanding for individuals and policy makers and supporting short-range decisions. To inform public health and support awareness of proper forecast interpretation when making decisions, it is important to understand the basics of the generation of forecasts and the boundaries under which their interpretations are valid. To this point, this study explores the error levels of seven common forecasting methods in estimating COVID-19 cumulative case counts at county, health district, and state levels over the upcoming week. Comparing error levels across forecasting methods and geographic granularities provides insight into the assumptions contributing to more accurate forecasts.

Small numbers of COVID-19 cases can lead to large outbreaks [7]. Isolation and preventative measures are recommended practices to reduce the spread of COVID-19 [1-3,8-14]. Forecasts with high error magnitudes can provide expectations that grossly underestimate or overestimate case counts. This can lead to problems, such as the creation of unanticipated hot spots resulting from underestimation, or can cause unnecessary public alarm from overestimation. Interpreting COVID-19 forecasts depends on assumptions such as the geographic area, preventative measures in place, and the population’s knowledge of, and behaviors toward, the virus. As assumptions change, the usefulness of the forecasting method should be re-evaluated. The impact of nonpharmaceutical interventions can be delayed 1 to 3 weeks and should factor into policy makers’ decisions [15]. Intervention methods can result in secondary effects, such as decreasing levels of physical activity while people practice social distancing [16]. As a result, understanding of the assumptions pertaining to short-range COVID-19 forecasting is needed to properly interpret their findings [17].

This study explores seven commonly utilized forecasting approaches, including the following: naïve [18], moving average (MA) [9,10], autoregressive (AR) [17], growth rate [19], Holt-Winters (HW) exponential smoothing [20,21], autoregressive moving average (ARMA) [22], and autoregressive integrated moving average (ARIMA) [23]. Each forecasting method utilizes different assumptions about how the past values impact the forecast values. The naïve approach is the simplest method and assumes no change from the current value. The MA approach assumes equal weighting of prior values, while exponential smoothing assigns exponentially decreasing weight to older values. The AR approach assumes linear dependency of prior values but with an added stochastic component. The growth rate approach assumes a linear relationship to its prior values and applies sampling with growth based on the number of increased cases from the prior day. The ARMA approach combines the AR approach to provide a regression based linearly on its past values with the MA approach to account for the error terms within the prior values. The ARIMA approach applies to data that is nonstationary around a mean value and applies a distancing measure one or more times to make the data stationary [24].

Error represents the inability to account for all the variability contributing to changes in COVID-19 case counts. Forecast error represents the under- or overestimation of the actual value [18]. Additionally, assumptions are unlikely to remain constant over time due to shifting public behaviors and implemented public policies. Error magnitude communicates the accuracy of a forecast and can be utilized as a metric to select from a set of potential forecasting methods. Interpreting forecast outcomes relies on the error magnitude as well as situating the assumptions underlining the forecast [25,26]. This means that the effectiveness of a current forecasting method is likely to be impacted as new preventative measures are put into place that alter spread dynamics. Conveying this understanding to the public advances knowledge of health statistics and statistical literacy in public health [21,27-29].

Recommendations for models of infectious diseases in support of public health involve incorporating policy questions, available data, and scientific understanding to yield policy advice, data collection, and scientific insight [30]. By evaluating forecasts by aggregating information from lower levels, such as at the county and health district levels, intervention strategies can be more readily applied based on the relevant demographic characteristics shared by the smaller population samples. Forecasting methods operate under differing assumptions pertaining to how the prior values relate to forecasted values. This study evaluates seven forecasting methods with varied look-back and forecast lengths at the county, health district, and state levels. By evaluating short-range forecasting methods combined with varied look-back and forecast lengths, forecasts can be more effectively used for informing health planning and aiding individuals in evaluating the safety levels of their local and neighboring communities.

## Methods

### Data

**Figure 1:**
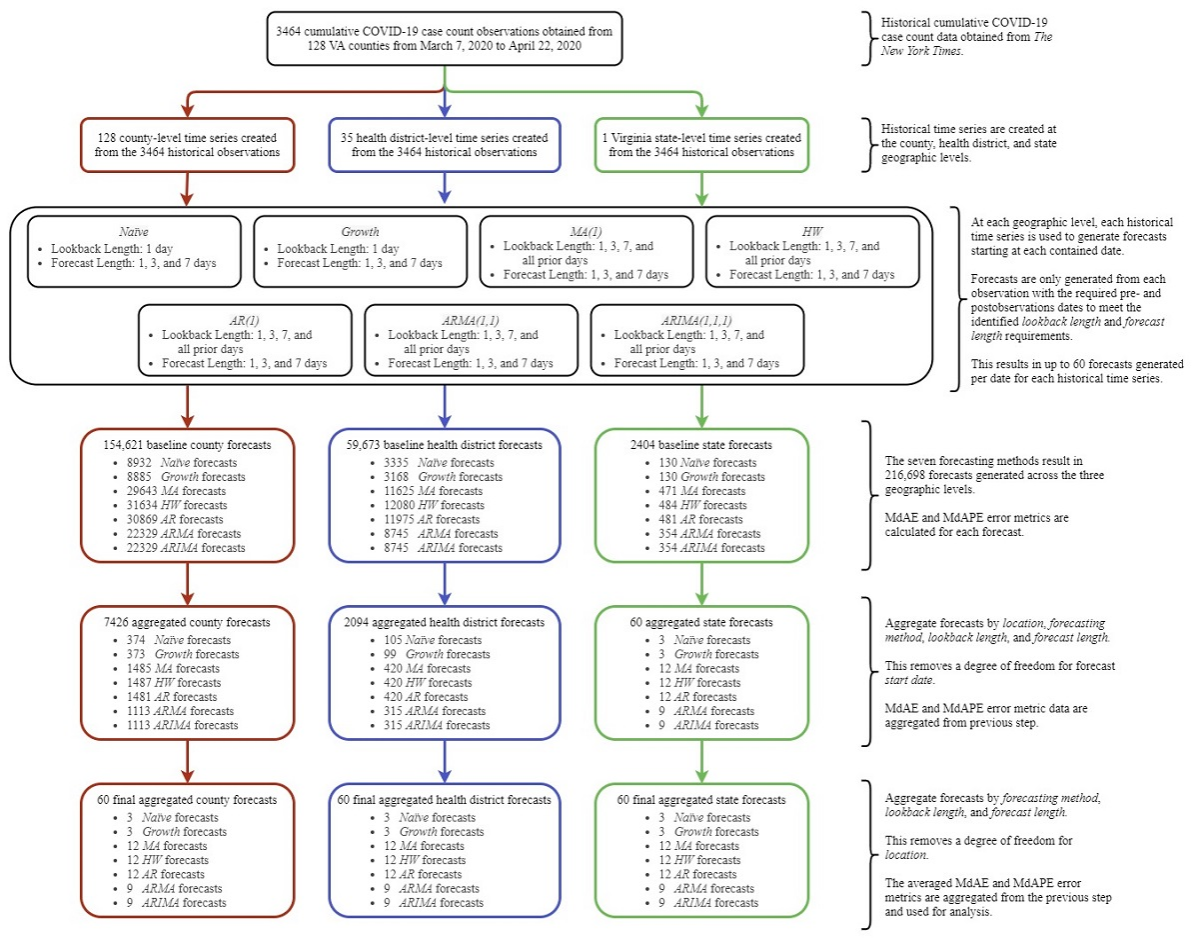
Experimental design and data overview at the county, health district, and state levels. The generation and aggregation of county-level forecasts are shown on the left path (red), health district–level forecasts on the middle path (blue), and state-level forecasts on the right path (green). The information on the right provides additional detail on each stage in the experimental design. AR: autoregressive; ARIMA: autoregressive integrated moving average; ARMA: autoregressive moving average; HW: Holt-Winters; MA: moving average; MdAE: median absolute error; MdAPE: median absolute percentage error; VA: Virginia.

We obtained 3464 Virginia county–level COVID-19 cumulative case count observations from March 7 to April 22, 2020, using data provided by *The New York Times* and aggregated these observations to the health district and state levels as presented in Figure 1. This period captured the first 3 weeks following the first confirmed COVID-19 case within Virginia and the 3 weeks following the governor’s executive order limiting gatherings to groups of less than 10 people. As intervention measures can take up to 3 weeks to impact the virus spread [15], this time frame was expected to cover Virginia’s case growth prior to experiencing the benefits resulting from the governor’s imposed group size limit.

### Forecasting Methods and Assumptions

For the naïve forecasts, the prior day’s value is used for each of the following *j* forecasted days. For the HW forecasts, exponential smoothing of the prior *k* day’s values is used to forecast values over the next *j* days. For the growth rate forecasts, the prior 1 day’s value is used to calculate the current growth rate over the following *j* days. Then, the prior day’s values for all the counties are used to calculate the growth rate for Virginia for the same *j* days. A group of *n* forecasts are generated for the county by uniformly sampling a growth rate between the county’s rates and Virginia’s rates. The average of the *n* forecasts is utilized as the final forecast for the county. For the MA (1), AR (1), ARMA (1, 1), and ARIMA (1, 1, 1) forecasts, the prior *k* days are given equal weighting to forecast the next *j* days.

This study only relies on the daily reported case numbers since the date of first onset within each location and does not incorporate assumptions about the basic reproductive number of COVID-19. Forecasts are influenced by the reliability of the data, the variables utilized, and the perceptions and reactions to danger and they assume the continuation of past patterns [4,31]. When exploring real-time forecasts of infectious disease models, real-time models have shown higher absolute error values, on average, than full-data models as a result of factors such as significant differences in population sizes between compared areas [32].

### Statistical Analysis

We aggregated median absolute error (MdAE) and median absolute percentage error (MdAPE) variables and expressed them as medians, IQRs, and notch ranges representing 95% CIs. Notch ranges are calculated as *±1.58 × IQR/√n* [33,34] as implemented using the R function geom_boxplot within the package ggplot2 (The R Foundation) [35]. Nonoverlapping comparisons of confidence intervals represent statistically significant differences [36,37] with *P* values less than .01 [38-41]. *P* values conveying significant differences between groups are calculated using Mood’s median test [42,43]. MdAE compared forecasting outcomes at shared geographic levels due to similarities in scale [44]. MdAPE compared each forecasting method’s outcomes across geographic level due to differing scales [45].

We created 226,468 forecasts across the county, health district, and state levels over the period of March 7 through April 22, 2020. Due to the naïve and growth rate methods only utilizing 1-day look-backs and the ARMA and ARIMA methods requiring more than 1-day look-backs, five forecast methods exist for each comparison. Analyses were performed with R software, version 3.6.3 (The R Foundation).

### Verification, Validation, and Reproducibility

The data set and code are provided in Lynch and Gore [46] and the experimental methods and steps needed for reproducibility are provided in Lynch and Gore [47]. Code inspections and unit tests were utilized for code verification [48]. MdAE and MdAPE error metrics were used for validation. A comparison of COVID-19 case count data sources found that the differences in reported case counts between *The New York Times*, *Johns Hopkins University*, and *USAFacts* do not indicate inferior or superior sources [49].

## Results

### Overview

Comparing all forecast methods’ MdAE values across the county, health district, and state levels over the first 46 days of infection revealed that MA forecasts using 3-day look-back and 1-day forecast length achieved the lowest MdAE. This MA forecast combination was statistically significantly different in MdAE  from 39 of the 59 other combinations at the county level (66 %),  53 of 59 (90%) at the health district level, and 51 of 59 (86%) at the state level. This result shows that the use of an equally weighted linear dependency with a stationary mean between the prior 3-day COVID-19 cumulative case counts, within the MA forecasts, is an effective assumption when forecasting next-day case growths for Virginia at the county, health district, and state levels. Table 1 provides the method with the lowest MdAE and the percentage of other methods from which the difference is determined to be statistically significant at the county level. Table 2 provides this information at the health district level and Table 3 provides this information at the state level.

For the methods using single-day look-back across all levels, the growth rate and naïve methods provided the lowest MdAE at the county, health district, and state levels for all forecast lengths. In general, all five methods achieved similar error confidence intervals when utilizing 1-day look-back. Only at the health district and state levels for 1-day forecast lengths was the growth rate method’s difference from the other methods statistically significant, with the growth rate method performing better than all combinations at the state level.

For the methods using 7-day look-backs across all levels, the MA and AR methods were the only ones with MdAE instances that were statistically significantly lower than the other methods. The HW and ARIMA methods achieved the lowest MdAE in two instances but did not perform significantly better than the other methods in either instance. In no instance did the ARMA method obtain the lowest MdAE. The performance of the MA and AR methods supports the assumption of linear dependence between the 7-day prior days’ cases and the forecast case counts. However, for the MA method the mean weighting of past values was stationary, while for the AR method it was nonstationary.

For the methods using look-backs of all prior-day case counts across all levels, the MA method achieved the lowest MdAE in all cases. This provides evidence in support of forecasting cumulative case counts using the assumption of a linear dependency and stationary mean among past values to forecast 1, 3, and 7 days when incorporating all prior cumulative cases.

**Table 1:** County-level median absolute error (MdAE) outcomes by forecasting method, look-back length, and forecast length.

Methods	Look-back length (days), n	Look-ahead length (days), n	df^a^	Forecasting method with lowest MdAE	Median (95% CI) (cumulative cases)	*P* value^b^	Statistically significantly lower MdAE than other methods^c^, n (%)
All (N=60)	All	All	59	MA (3, 1)^d^	0.67 (0.49-0.84)	<.001	39 (66)
G1^e^ (n=5)	1	1	4	Naïve	0.67 (0.43-0.90)	.09	0 (0)
G1 (n=5)	1	3	4	Naïve	1.30 (0.88-1.73)	.66	0 (0)
G1 (n=5)	1	7	4	Naïve	2.43 (1.69-3.18)	.50	0 (0)
G2^f^ (n=5)	3	1	4	MA	0.67 (0.49-0.84)	.09	0 (0)
G2 (n=5)	3	3	4	MA	0.76 (0.59-0.94)	<.001	4 (100)^g^
G2 (n=5)	3	7	4	MA	1.69 (1.36-2.01)	<.001	3 (75)
G2 (n=5)	7	1	4	HW	0.91 (0.63-1.18)	.03	0 (0)
G2 (n=5)	7	3	4	MA	1.30 (0.95-1.65)	.002	1 (25)
G2 (n=5)	7	7	4	MA	2.32 (1.75-2.90)	.01	0 (0)
G2 (n=5)	All prior	1	4	MA	0.70 (0.53-0.87)	.33	0 (0)
G2 (n=5)	All prior	3	4	MA	0.83 (0.67-1.00)	<.001	4 (100)^g^
G2 (n=5)	All prior	7	4	MA	1.73 (1.36-2.10)	<.001	1 (25)

^a^Degrees of freedom represent the number of forecasting combinations minus one.

^b^*P* values were calculated for statistically significant differences in medians across groups.

^c^This was based on comparisons of notch ranges. MdAE was interpreted within geographic levels.

^d^MA: moving average; (3, 1) represents a 3-day look-back and a single-day forecast length.

^e^G1 includes naïve, MA, autoregressive (AR), growth rate, and Holt-Winters (HW) methods.

^f^G2 includes MA, AR, growth rate, HW, autoregressive moving average, and autoregressive integrated moving average methods.

^g^MA (3, 3) and MA (all prior, 3) achieved statistically significantly smaller MdAE than all four alternatives.

**Table 2:** Health district–level median absolute error (MdAE) outcomes by forecasting method, look-back length, and forecast length.

Methods	Look-back length (days), n	Look-ahead length (days), n	df^a^	Forecasting method with lowest MdAE	Median (95% CI) (cumulative cases)	*P* value^b^	Statistically significantly lower MdAE than other methods^c^, n (%)
All (N=60)	All	All	59	MA (3, 1)^d^	3.07 (2.41-3.74)	<.001	53 (90)
G1^e^ (n=5)	1	1	4	Growth rate	4.03 (3.01-5.04)	.31	1 (25)
G1 (n=5)	1	3	4	Growth rate	8.96 (6.56-11.36)	.93	0 (0)
G1 (n=5)	1	7	4	Growth rate	16.48 (11.67-21.28)	.96	0 (0)
G2^f^ (n=5)	3	1	4	MA	3.07 (2.41-3.74)	.01	1 (25)
G2 (n=5)	3	3	4	MA	3.20 (2.50-3.90)	<.001	4 (100)^g^
G2 (n=5)	3	7	4	MA	7.88 (5.71-10.05)	<.001	1 (25)
G2 (n=5)	7	1	4	AR	3.57 (2.67-4.47)	.01	0 (0)
G2 (n=5)	7	3	4	MA	5.52 (3.96-7.08)	<.001	2 (50)
G2 (n=5)	7	7	4	AR	11.83 (8.16-15.49)	<.001	1 (25)
G2 (n=5)	All prior	1	4	MA	3.14 (2.47-3.80)	.04	1 (25)
G2 (n=5)	All prior	3	4	MA	3.16 (2.54-3.78)	<.001	3 (75)
G2 (n=5)	All prior	7	4	MA	7.68 (6.22-9.14)	<.001	3 (75)

^a^Degrees of freedom represent the number of forecasting combinations minus one.

^b^*P* values were calculated for statistically significant differences in medians across groups.

^c^This was based on comparisons of notch ranges. MdAE was interpreted within geographic levels.

^d^MA: moving average; (3, 1) represents a 3-day look-back and a single-day forecast length.

^e^G1 includes naïve, MA, autoregressive (AR), growth rate, and Holt-Winters (HW) methods.

^f^G2 includes MA, AR, growth rate, HW, autoregressive moving average, and autoregressive integrated moving average methods.

^g^MA (3, 3) achieved statistically significantly smaller MdAE than all four alternatives.

**Table 3:** State-level median absolute error (MdAE) outcomes by forecasting method, look-back length, and forecast length.

Methods	Look-back length (days), n	Look-ahead length (days), n	df^a^	Forecasting method with lowest MdAE	Median (95% CI) (cumulative cases)	*P* value^b^	Statistically significantly lower MdAE than other methods^c^, n (%)
All (N=60)	All	All	59	MA (3, 1)^d^	17.43 (7.74-27.11)	<.001	51 (86)
G1^e^ (n=5)	1	1	4	Growth rate	31.50 (6.11-56.89)	<.001	4 (100)^f^
G1 (n=5)	1	3	4	Growth rate	317.50 (163.15-471.85)	.94	0 (0)
G1 (n=5)	1	7	4	Growth rate	325.00 (169.49-480.51)	.18	0 (0)
G2^g^ (n=5)	3	1	4	MA	17.43 (7.74-27.11)	<.001	4 (100)^f^
G2 (n=5)	3	3	4	MA	64.94 (45.93-83.96)	<.001	1 (25)
G2 (n=5)	3	7	4	MA	206.57 (148.57-264.94)	.03	1 (25)
G2 (n=5)	7	1	4	AR	69.37 (34.23-104.51)	.09	1 (25)
G2 (n=5)	7	3	4	MA	82.14 (42.83-121.47)	.02	2 (50)
G2 (n=5)	7	7	4	ARIMA	312.36 (146.54-478.17)	.012	0 (0)
G2 (n=5)	All prior	1	4	MA	25.13 (11.61-38.65)	.27	0 (0)
G2 (n=5)	All prior	3	4	MA	32.67 (21.20-44.14)	.002	2 (50)
G2 (n=5)	All prior	7	4	MA	104.85 (70.67-139.03)	.09	2 (50)

^a^Degrees of freedom represent the number of forecasting combinations minus one.

^b^*P* values were calculated for statistically significant differences in medians across groups.

^c^This was based on comparisons of notch ranges. MdAE was interpreted within geographic levels.

^d^MA: moving average; (3, 1) represents a 3-day look-back and a single-day forecast length.

^e^G1 includes naïve, MA, autoregressive (AR), growth rate, and Holt-Winters (HW) methods.

^f^Growth rate (1, 1) and MA (3, 1) achieved statistically significantly smaller MdAE than all four alternatives.

^g^G2 includes MA, AR, growth rate, HW, autoregressive moving average, and autoregressive integrated moving average (ARIMA) methods.

### County-Level MdAE Results

**Figure 2:**
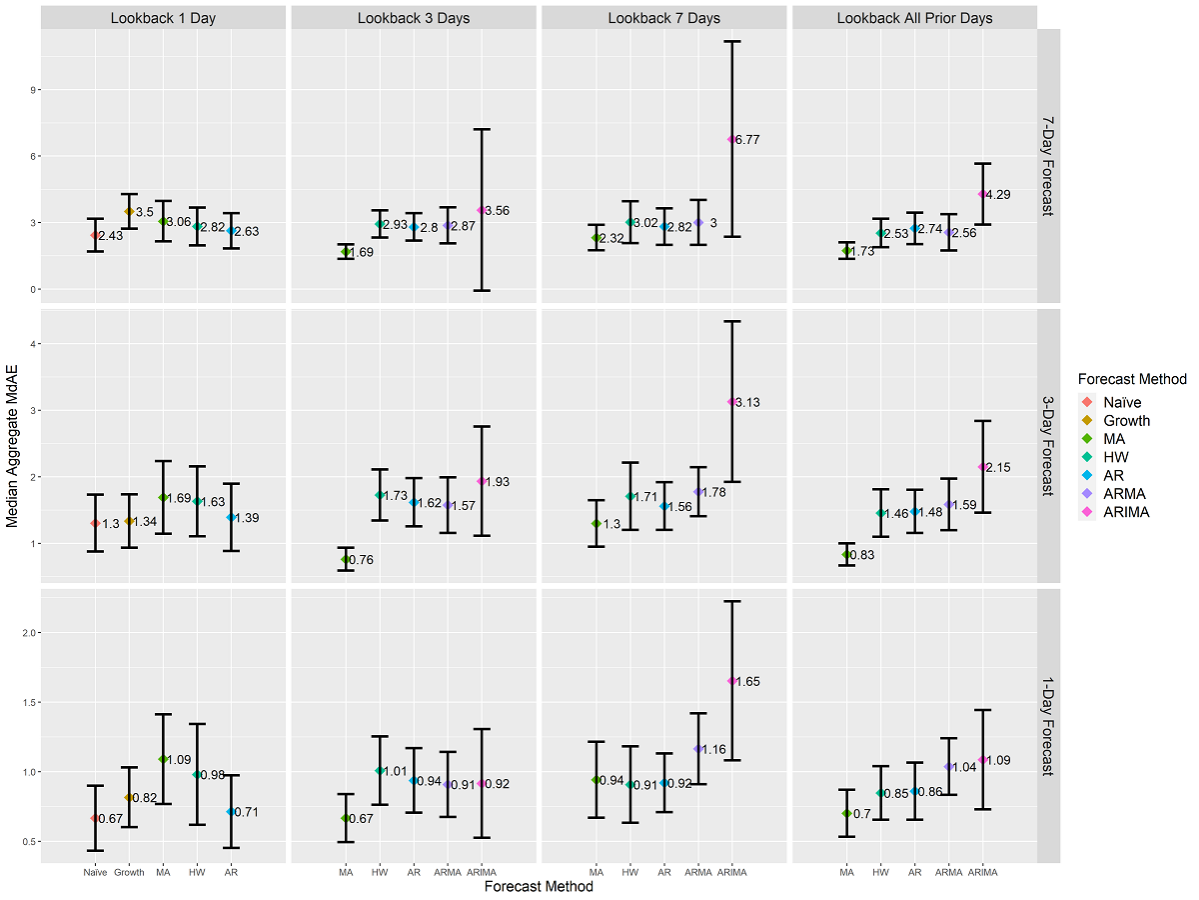
County-level forecasts’ aggregated median MdAE values and 95% CI. CI ranges are calculated using box plot notch ranges around the median. Statistically significant differences at a *P* value of .01 are identified by nonoverlapping CI ranges of forecasting methods at each combination of forecast length and look-back length. Units are in terms of COVID-19 cumulative case counts. Y-axis scales differ on each row based on the scale of the contained data. Due to differing assumptions, five of the seven forecasting methods are present for each look-back length as indicated on the x-axis. AR: autoregressive; ARIMA: autoregressive integrated moving average; ARMA: autoregressive moving average; HW: Holt-Winters; MA: moving average; MdAE: median absolute error.

At the county level, the MA method always achieved a lower MdAE than the ARMA method. Similarly, the ARMA method always achieved a lower MdAE than the ARIMA method. Thus, the ARIMA method’s aggregated error was greater than the ARMA method’s aggregated error, which was greater than the MA method’s aggregated error. This indicates that the assumption of a stationary mean (ie, MA) in prior case counts is more effective than the assumption of a weakly stationary mean (ie, ARMA), which is more effective than a nonstationary mean (ie, ARIMA) when forecasting at the county level. The ARIMA method had the widest confidence interval for the median error range, indicating the least consistency in COVID-19 forecasts among these methods. Figure 2 provides the county-level MdAE outcomes for each look-back and forecast length combination. The individual results of each of the 60 forecasting combinations at the county level are provided in Multimedia Appendix 1, including median values, confidence intervals, whiskers, sample sizes, and *P* values. An interactive version of Figure 2 is provided in Multimedia Appendix 2.

### Health District–Level MdAE Results

**Figure 3:**
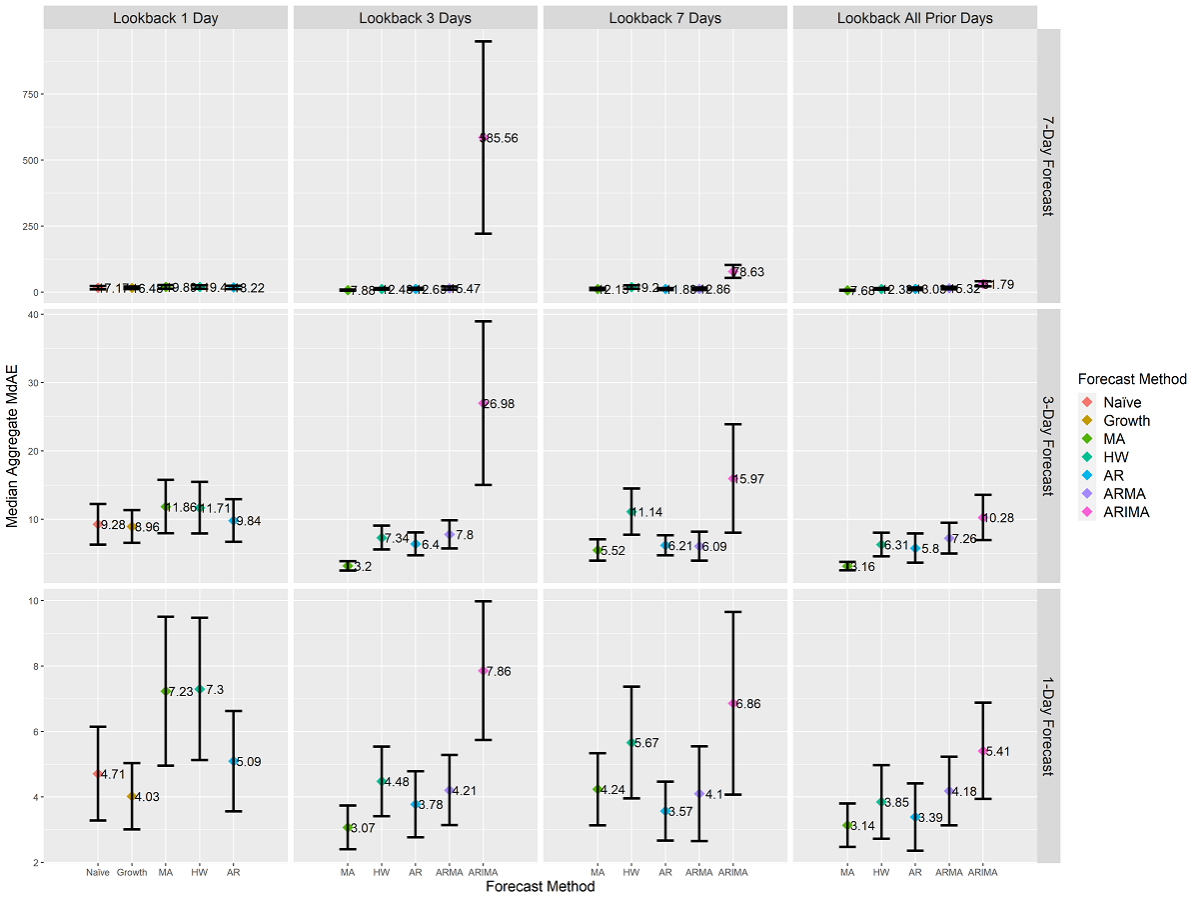
Health district–level forecasts’ aggregated median MdAE values and 95% CI. CI ranges are calculated using box plot notch ranges around the median. Statistically significant differences at a *P* value of .01 are identified by nonoverlapping CI ranges of forecasting methods at each combination of forecast length and look-back length. Units are in terms of COVID-19 cumulative case counts. Y-axis scales differ on each row based on the scale of the contained data. Due to differing assumptions, five of the seven forecasting methods are present for each look-back length as indicated on the x-axis. AR: autoregressive; ARIMA: autoregressive integrated moving average; ARMA: autoregressive moving average; HW: Holt-Winters; MA: moving average; MdAE: median absolute error.

At the health district level, the MA method always achieved lower MdAE than the ARMA method, which achieved lower MdAE than the ARIMA method. This further provided evidence that effective forecasting of cumulative COVID-19 case counts contains an assumption of stationary means in past observations. For 3-day look-back lengths with 3-day forecasts, the MA method achieved statistically significantly lower MdAE than all other methods. Figure 3 provides the MdAE at the intersection of look-back length and forecast length at the health district level. The individual results of each of the 60 forecasting combinations at the health district level are provided in Multimedia Appendix 3, including median values, confidence intervals, whiskers, sample sizes, and *P* values. An interactive version of Figure 3 is provided in Multimedia Appendix 4.

### State-Level MdAE Results

**Figure 4:**
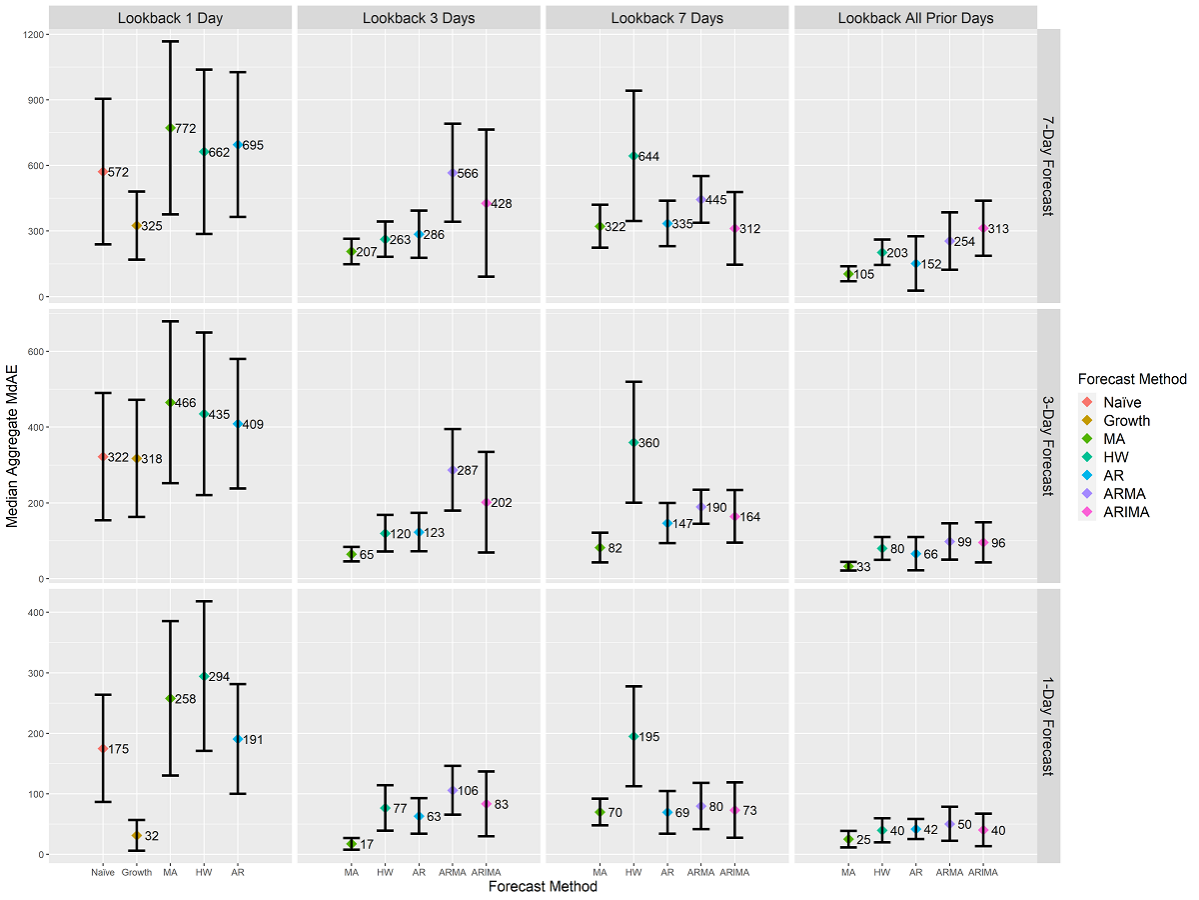
State-level forecasts’ aggregated median MdAE values and 95% CI. CI ranges are calculated using box plot notch ranges around the median. Statistically significant differences at a *P* value of .01 are identified by nonoverlapping CI ranges of forecasting methods at each combination of forecast length and look-back length. Units are in terms of COVID-19 cumulative case counts. Y-axis scales differ on each row based on the scale of the contained data. Due to differing assumptions, five of the seven forecasting methods are present for each look-back length as indicated on the x-axis. AR: autoregressive; ARIMA: autoregressive integrated moving average; ARMA: autoregressive moving average; HW: Holt-Winters; MA: moving average; MdAE: median absolute error.

At the state level, the growth rate method was the most effective method. In every case, it either (1) attained the lowest MdAE value compared to the other methods or (2) had the smallest notch range. The ARMA and ARIMA methods both maintained MdAE notch bands that were similar to the other methods when utilizing all prior day and 7-day look-back lengths. However, the HW method’s MdAE notch bands increased as that of the ARMA and ARIMA methods decreased. These results make it unclear as to which of the assumptions related to stationary means were most effective for forecasting with the ARMA and ARIMA methods at the state level. Figure 4 provides MdAE values at the intersection of look-back length and forecast length at the state level. The individual results of each of the 60 forecasting combinations at the state level are provided in Multimedia Appendix 5, including median values, confidence intervals, whiskers, sample sizes, and *P* values. An interactive version of Figure 4 is provided in Multimedia Appendix 6.

### Cross-Geographic-Level MdAPE Results

**Figure 5:**
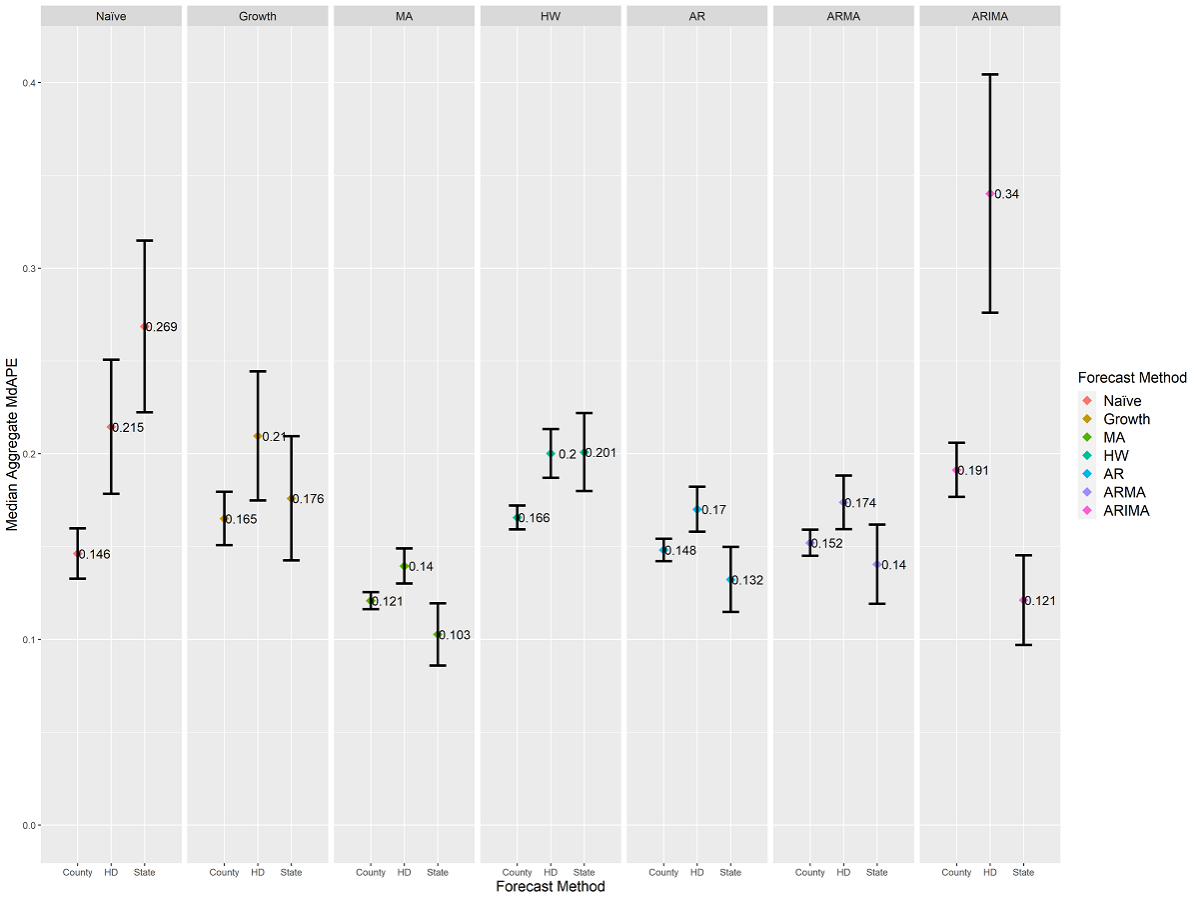
Aggregated median MdAPE values and 95% CI ranges at the county, health district (HD), and state levels differentiated by forecasting method. Comparing CI ranges for a forecast method across each geographic level reveals statistically significant differences in median values for the forecasting method due to geographic scale. Nonoverlapping CI ranges indicate statistically significant differences at a *P* value of .01. MdAPE provides a comparison within each forecast method separately, not a comparison across different methods. AR: autoregressive; ARIMA: autoregressive integrated moving average; ARMA: autoregressive moving average; HW: Holt-Winters; MA: moving average; MdAPE: median absolute percentage error.

MdAE reflects the scale of the data and is not appropriate for making inferences about changes in confirmed case counts between county, health district, and state levels [26,44]. Figures 2-4 convey differing scales of error values across the three levels. As a result, it was not possible to evaluate results featured in these figures against each other. To remedy this shortcoming, we applied MdAPE to identify statistically significant differences for each forecasting method individually when applied to county, health district, and state levels as provided in Figure 5. The individual results of each of the 60 forecasting combinations at the county, health district, and state levels are provided in Multimedia Appendices 7-9, including median values, confidence intervals, whiskers, sample sizes, and *P* values. An interactive version of Figure 5 is provided in Multimedia Appendix 10.

Statistically significant differences were observable within a forecasting method across the county, health district, and state levels during the initial 46 days of confirmed COVID-19 case spread within Virginia. The growth rate method was the only one whose performance did not statistically significantly differ across levels; thereby, it was the only method unaffected by geographic level. The naïve method achieved a statistically significantly lower MdAPE at the county level than at the health district and state levels. The MA, HW, AR, ARMA, and ARIMA methods all contained instances of achieving statistically significantly lower MdAPE scores at the county and/or state levels than at the health district level.

## Discussion

### Principal Findings

Our results show the effectiveness of seven forecasting methods for the first 46 days of virus spread within Virginia at the county, health district, and state levels. In addition, a daily view of the growth rate forecast at the county level from March 7, 2020, to the present is publicly available online [50]. Tracking case and death counts yield insight into the virus’s impact on a geographic region at a given point in time. Forecasts utilize the trends and averages of prior case count observations to provide expectations of case counts into the future. These forecasts keep the public informed on the state of the virus across the world and on virus levels within their own geographic areas of interest. Additionally, forecasts inform public policy for combatting the spread of the virus, supporting public health, and helping to anticipate the impacts of medical burdens across regions [51]. However, interpreting forecast outcomes requires understanding the assumptions behind the forecasting method as well as the assumptions pertaining to the geographic area and the presence of intervention strategies. Therefore, we compared the error levels pertaining to 60 forecasting combinations using the MA, AR, naïve, growth rate, HW, ARMA, and ARIMA forecasting methods. Our findings support public health with respect to forecasting by reinforcing health statistics and statistical literacy of forecasted COVID-19 outcomes.

COVID-19 cumulative case growth is such that the growth curve is exponential in the absence of preventative measures. The larger error observed in HW forecasts over MA forecasts provides support that an exponential model is not the best fit at the start of the virus spread for Virginia. The preventative measures taken by the population appear to have shifted the virus’s growth behavior from exponential to linear. This finding is also supported by Lammers et al [21]. This finding supports the idea that population interventions are effective at impacting the spread of the virus. However, as the virus continues to spread and reoccur, the inability to manufacture a vaccine for COVID-19 quickly enough to immunize the population remains a concern [52]. As such, combining short-range forecasts at the county and health district levels with targeted intervention strategies can improve planning, support, and response time. The use of rigorous government interventions may slow the rate of infections, but early detection, isolation, treatment, and adequate medical supplies are required for continued intervention against the virus [53,54].

Our cross-geographic validation checks using MdAPE indicate that the level of geographic resolution should be considered when creating forecasts of expected case counts. A forecast utilized at the state level is not likely to be as useful for determining expected growth when disaggregated across its counties during early virus onset. This results from the differing geographic assumptions present within counties or health districts when compared to the state. This finding is consistent with the literature reporting that case growths vary across countries and across states [14]. Variations result from factors such as population behaviors in response to the pandemic, implemented policy interventions, and population densities [7,9,10,15]. Furthermore, since the growth rate method did not produce statistically significant MdAPE differences across geographic levels, it may be a good choice for decision makers whose region does not match the county, health district, or state levels.

To identify a best option among our tested combinations, we compared MdAE ranges against each other within each geographic tier. The MA method using a 3-day look-back length and a single-day forecast length provided the smallest error (ie, lowest MdAE) at the county level (median 0.67, 95% CI 0.49-0.84; *P*<.001), the health district level (median 3.07, 95% CI 2.41-3.74; *P*<.001), and the state level (median 17.43, 95% CI 7.74-27.11; *P*<.001). Compared to the other forecasting combinations, the MA method’s confidence intervals statistically significantly differed from 39 out of 59 alternatives (66%; county level) to 51 out of 59 alternatives (86%; state level) to 53 out of 59 alternatives (90%; health district level) at a *P* value level of .01. When relying on only the prior day’s case counts, the growth rate method stood out as the best option at the health district and state levels; however, the naïve, growth rate, HW, MA, and AR methods performed similarly well at the county level.

When utilizing 3 or more days of prior observations, a diverse range of options is available. For next-day forecasts, there was no method that performed better at a level that was statistically significant among the five options. For 3-day forecasts, the MA method was statistically significantly better than 25% to 100% of the other four options in all cases. For 7-day forecasts, the MA method performed statistically significantly better than 25% to 75% of the other four options when using a 3-day look-back or an all prior–day look-back. When using a 7-day look-back, the AR method performed the best and its difference from the ARIMA method was statistically significant. The ARMA and ARIMA methods achieved the lowest error in any of the combinations.

These findings support the assumption of stationarity within the mean of the prior days’ cumulative case counts. This is reflected in how well the MA method performed and how poorly the ARIMA method performed at forecasting cumulative case counts. Rarely do the ARIMA or ARMA methods achieve lower error values than any other combination. This reflects the idea that the assumption of stationary means of past observations is a more effective representation of cumulative COVID-19 growth than assumptions of weak stationarity or nonstationarity. The need to apply a differencing step to remove nonstationarity using the ARIMA method is not present within the data during this period. Additionally, placing extra weight on the recent past does not improve forecasting during this period, as the HW and AR methods were consistently less effective than the MA method. These findings suggest that the ARIMA and ARMA methods are unlikely to be good fits and should not be used to forecast case counts during early onset within areas that have only a few weeks of historical data collected, whose residents are aware of the existence of the virus and are engaging in preventative behaviors, and that contain similar population densities to Virginia.

Several studies utilized forecasts to estimate case fatality and recovery ratios, epidemiological parameters, and transmission dynamics based on data from the start of the outbreak [55,56]. Studies also support the idea that epidemiological differences contribute to variations in the severity of the contracted disease [2,57]. Based on historical similarities to previous influenza strains, social distancing can potentially reduce transmission of the virus; however, the effectiveness may vary alongside changes in seasonal factors in travel as well as between tropical and temperate climates [58,59]. Distancing may be especially beneficial in rural areas, where fewer hospitals and health care facilities exist, by emphasizing strategies oriented toward specific population age groups [60].

The results of this study can be expanded to include areas’ demographic characteristics, geographic characteristics, and preventative measures to strive for more accurate forecast models. A recent study found COVID-19 growth to strongly correlate with population density, percent of the population living in rural areas, and yearly flu vaccination rate [14]. Exploring forecast behaviors of areas sharing these traits may further reduce forecasting error and reveal subgroupings of viable forecasting method options. Additionally, forecasting models can be paired with mortality models [61] to gain better estimates of infection forecasts per demographic characteristics. Predictive methods derived from search engines’ data [62] can also be incorporated within forecasting methods. This would provide a way to connect forecasts with human search behaviors based on the frequency of searched terms identified in relation to COVID-19 prevention and recovery. Forecasting models can be paired with models of local medical burden and pandemic preparedness [51] for more detailed representations of expected medical strains and greater flexibility in testing preventative measures.

Stay-at-home orders have been successful as intervention strategies to slow the spread of the virus. However, the impact caused by a neighboring area’s removal of mobility restrictions needs greater exploration [10]. The secondary effects of the starting or ending of proximity-based prevention methods on neighboring areas can help assess the potential impact of a mitigation strategy. For identified hot spots, the Centers for Disease Control and Prevention provides outreach to local officials and helps in identifying adapted interventions for the local area [7]. Rapid identification and timeliness of response are critical, especially if the impact of interventions can take up to 3 weeks to be effective [15]. Reliable forecasting can aid in the identification of emerging hot spots and support timely response. To this end, increased knowledge of forecasting characteristics based on geographic level, demographic characteristics, population density characteristics, and population behaviors can help reveal the primary drivers of upcoming cases. This knowledge can be leveraged to inform early, targeted interventions or to provide risk updates to targeted populations within an area. People could then modify their mobility and social decisions themselves in a timely manner separate from population mandated measures.

### Limitations

Here we discuss internal and external validity threats as well as other limitations that affected our work. Internal validity threats arise when factors affect the dependent variables without the researchers’ knowledge. It is possible that some implementation flaws could have affected our modeling results or the ensuing data analysis. However, the algorithms in our source code were (1) built on established libraries, (2) passed several internal code reviews, and (3) are publicly accessible, along with the data and results. Threats to external validity occur when the results of our analysis and our simulation cannot be generalized. Our results are limited to Virginia, from March to April 2020 with respect to COVID-19 cases reported by *The New York Times*. Our results are not immediately generalizable to (1) different infectious diseases, (2) other COVID-19 data sets, (3) different periods of time, or (4) different geographic areas.

Several other assumptions and limitations pertain to this study. Seven forecasting methods with differing baseline assumptions were evaluated with respect to how well they forecast the early growth of COVID-19 cases within Virginia; however, numerous additional forecasting methods exist with different combinations of assumptions that can also be explored with respect to this pandemic. Conclusions should not be drawn about the effectiveness of these findings for forecast lengths greater than 7 days, as the appropriateness of underlying assumptions, such as stationarity of prior days’ values, would need to be re-evaluated. Larger median error values of the 7-day forecasts, versus their 1- and 3-day counterparts, were observable, further supporting the need for evaluation of forecast assumptions pertaining to the characteristics of COVID-19 beyond 7-day forecast lengths. The selected forecasting methods assumed that policies and population behaviors remained unchanged during the forecast periods; therefore, the forecasts do not account for future starts or ends of policies, such as stay-at-home orders or return-to-work dates.

Finally, our results do not reflect how the spread of the virus would occur for locations experiencing first contact with the virus without yet having public awareness of the existence of the virus. These findings are applicable under the assumption that the general population was already aware of the presence of COVID-19. At the starting point of Virginia cases, the local population was already aware that cases had reached the United States, the virus had been classified as a pandemic, and the virus was receiving major media attention. Additionally, the governor had issued an executive order declaring a state of emergency due to COVID-19 on March 12, 2020 [10,63]. This provided 47 days for Virginians to prepare and modify their standard movement and interaction behaviors as they deemed necessary for their own safety. As a result, these results were not captured in the same context as the period of time when areas within the United States were first impacted by the virus (ie, areas of Washington, California, and New York).

## Appendix

Multimedia Appendix 1 County-level median absolute error (MdAE) outcomes for each forecasting combination.

Multimedia Appendix 2 Interactive plot conveying county-level median absolute error (MdAE) outcomes for each forecasting combination.

Multimedia Appendix 3 Health district–level median absolute error (MdAE) outcomes for each forecasting combination.

Multimedia Appendix 4 Interactive plot conveying health district–level median absolute error (MdAE) outcomes for each forecasting combination.

Multimedia Appendix 5 State-level median absolute error (MdAE) outcomes for each forecasting combination.

Multimedia Appendix 6 Interactive plot conveying state-level median absolute error (MdAE) outcomes for each forecasting combination.

Multimedia Appendix 7 County-level median absolute percentage error (MdAPE) outcomes for each forecasting combination.

Multimedia Appendix 8 Health district–level median absolute percentage error (MdAPE) outcomes for each forecasting combination.

Multimedia Appendix 9 State-level median absolute percentage error (MdAPE) outcomes for each forecasting combination.

Multimedia Appendix 10 Interactive figure conveying county-, health district–, and state-level median absolute percentage error (MdAPE) outcomes for each forecasting combination.

## Abbreviations

AR: autoregressive
ARIMA: autoregressive integrated moving average
ARMA: autoregressive moving average
HW: Holt-Winters
MA: moving average
MdAE: median absolute error
MdAPE: median absolute percentage error
